# Post-discharge quality of life of COVID-19 patients at 1-month follow-up: A cross-sectional study in the largest tertiary care hospital of Bangladesh

**DOI:** 10.1371/journal.pone.0280882

**Published:** 2023-01-31

**Authors:** Mohammad Mahfuzul Hoque, Ponkaj Kanti Datta, Kamalesh Chandra Basu, Muhammad Faizur Rahman, Mohammed Masudul Hassan Khan, Mohammad Mostafa Kamal, Reaz Mahmud, Kazi Ali Aftab, Ejrarul Alam Khan, Imran Mahmud, Rumana Sharmin, Md. Abdullah Saeed Khan, Mohammad Jahid Hasan, Md. Robed Amin, Md. Titu Miah, Md. Mujibur Rahman

**Affiliations:** 1 Department of Medicine, Dhaka Medical College, Dhaka, Bangladesh; 2 Department of Neurology, Dhaka Medical College, Dhaka, Bangladesh; 3 Department of Endocrinology, Dhaka Medical College, Dhaka, Bangladesh; 4 Department of Pathology, National Institute of Cancer Research & Hospital, Dhaka, Bangladesh; 5 Pi Research Consultancy Center, Dhaka, Bangladesh; Universitas Padjadjaran, INDONESIA

## Abstract

There is increasing evidence of the post-COVID-19 suffering and decreased quality of life in the COVID-19 patients. This study aimed to assess the quality of life and associated factors of COVID-19 patients at one month after discharge from the hospital. This was a cross-sectional study that was conducted at the post-covid clinic of Dhaka Medical College Hospital (DMCH) where RT–PCR-confirmed adult COVID-19 recovered patients were enrolled one month after discharge from the same hospital. They were consecutively selected from January 01 to May 30. A pretested semi-structured questionnaire was used for the data collection for clinical variables. The generic multi-attributable utility instrument EQ-5D-5L was used for assessing health-related quality of life (HRQoL). A total of 563 patients were enrolled in the study. The patients had a mean age with standard deviation (±SD) of 51.18 (±13.49) years and 55.95% were male. The mean (SD) EQ-5D-5L index score and EQ-VAS scores were 0.78 (±0.19) and 70.26 (±11.13), respectively. Overall, 45.77%, 50.99%, 52.79%, 55.14% and 62.16% had problems (slight to extreme) in the mobility, self-care, usual activities, pain/discomfort and anxiety/depression dimensions, respectively. Patients aged ≥60 years had significant problem in mobility (odds ratio [OR] 3.24, 95% confidence interval [CI]: 1.07–9.77). Female participants were 5.50 times (95% CI: 2.22–13.62) more likely to have problems in their usual activities. In comparison to urban area, living in a peri-urban setting was significantly associated with problems in mobility (OR 1.89, 95% CI: 1.13–3.20), pain/discomfort (OR 1.82, 95% CI: 1.04–3.12) and anxiety/depression (OR 2.16, 95% CI: 1.22–3.84). Comorbid patients were 1.75 times (95% CI: 1.07–2.85) more likely to report problems in the pain/discomfort dimension. Presence of symptom(s) was associated with problems in self-care (OR 3.27, 95%CI: 1.31–8.18), usual-activity (OR 3.08, 95%CI: 1.21–7.87), pain/discomfort dimensions (OR 2.75, 95%CI: 1.09–6.96) and anxiety/depression (OR 3.35, 95%CI: 1.35–8.30). Specific management strategies should be planned to address the factors associated with low health-related quality of life in post-acute care of COVID-19 patients.

## Introduction

Globally, coronavirus disease-19 (COVID-19) has affected nearly 289 million people and killed over 5.4 million by the end of 2020 [1]. In Bangladesh, over 1.5 million people were affected, and just over 28 thousand people died due to COVID-19 as of December 2021 [2]. Despite widespread vaccination around the world, new variants such as Delta and Omicron are still infecting a large number of people and continue to impede their lives [3].

The COVID-19 pandemic and the drastic measures taken to control it affected people in all spheres of life, thereby, exerted negative effect in their quality of living. It not only affected them physically, but also psychologically [4], socially [5], and economically [6]. Because of the fear of becoming infected, disruption of daily activities, impairment of social lives and economic uncertainty, emotional distress and psychiatric illnesses were widespread [7]. In Bangladesh, a high level of depression, anxiety, and stress was reported among COVID-19 patients and the healthy general population [8–11] and a substantial impact on their economic activity was reported [8].

Amid this widespread uncertainty and challenging environment, returning to normal was the most difficult for those who contracted the disease and recovered from it. Some patients remain physically ill in the form of muscle weakness and fatigue six months after recovery and harbor a high risk of developing anxiety and depression [12]. Globally, a considerable reduction in the health-related quality of life (HRQoL) of patients with acute and long COVID was documented [13].

In two recent population-based cross-sectional studies, the quality of life (QoL) of COVID-19 recovered patients in Bangladesh measured by the World Health Organization Quality of Life instrument brief version (WHOQOL BREF) [14] and Center for Disease Control and Prevention Health Related Quality of Life instrument (CDC HRQOL-14) were found to be significantly affected in multiple dimension [15, 16]. The former study reported a significantly lower QoL in physical, psychological and social dimensions of those with a history of hospitalization. Hospitalized COVID-19 patients were found to experience a substantial impairment in HRQoL after discharge in many other countries [17–20]. Hence, it is essential to explore the HRQoL and associated factors of hospitalized COVID-19 patients after discharge, so that tailored interventions can be taken to aid their recovery to full physical and mental health [16]. However, in Bangladesh, HRQoL assessment of COVID-19 patients short time after discharge from hospital is scarce. Hence, this study aimed to determine the HRQoL of post-acute-care COVID-19 patients one month after discharge from the largest tertiary care hospital of the country using the EQ-5D-5L instrument.

## Materials and methods

### Study participants, settings, and design

This cross-sectional study was conducted in the COVID-19 Post-acute Care and Follow-up Clinic of Dhaka Medical College Hospital (DMCH). DMCH is the largest tertiary care public hospital of Bangladesh [21], which has dedicated beds for the management of COVID-19 patients [22]. RT–PCR-confirmed COVID-19 adult patients (age ≥ 18 years) who were hospitalized during the acute illness and were discharged with recovery or with consent for home-based treatment after reduction in severity were targeted. Those who came for post-acute care one month after discharge between January 2021 and May 2021 were approached for enrollment. Recovery was defined by either a negative RT-PCR test and/or absence of fever, cough, and shortness of breath for at least three consecutive days before discharge. Informed written consent was obtained before inclusion. Patients who declined to enroll were excluded. A total of 594 post-acute-care COVID-19 patients were enrolled during the study period. Among them, 31 patients had incomplete data and were excluded. Finally, data from a total of 563 patients were analyzed.

### Data collection instrument

All the participants were interviewed by a pretested semi-structured questionnaire to determine sociodemographic information, clinical information during follow-up, and health-related quality of life (HR-QoL) measured during the first visit one month after discharge by the validated Bangla version of the EQ-5D-5L instrument [23].

### Sociodemographic, anthropometric, and lifestyle-related information

The sociodemographic section of the questionnaire obtained information on the patient’s age, sex (male/female), occupation, and settings (urban/rural/peri-urban). Peri-urban area was defined as areas proximate to urban context which maintained traditional institutional arrangements derived from the cultures of original residents for a considerable period of time [24]. Questions recording information on weight, height, Body Mass Index (BMI), lifestyle, and smoking habits were also included.

### Clinical characteristics and comorbidities

To assess the condition of patients during follow-up, a list of clinical features was included after reviewing the published literature. The clinical features considered were fever, fatigue, anorexia, weight loss, dyspnea, chest pain, cough, palpitation, hemoptysis, headache, dysgeusia, dysosmia, altered consciousness, sleep disturbance, dizziness, vertigo, weakness, symptoms of cranial nerve involvement and sensory disturbance, seizure, psychiatric symptoms (anxiety, depression, delusion, hallucination), vomiting, alternation of bowel habit, abdominal pain, joint pain, body/calf pain/limb pain, skin rash, loss of hair, and any other symptoms. Moreover, pulse, blood pressure, temperature, and SpO_2_ at rest were recorded at the time of interview. Information on comorbidities was collected from medical documents of patients.

### EQ-5D-5L

There are many multi-attributable utility instrument (MAUI) which can be used in the assessment of HRQoL [25], including the widely used WHOQOL BREF [14], Short Form (SF)-36 [26], CDC HRQOL-14 [27] and EQ-5D [23] instruments. Among these, the EQ-5D-5L instrument, a generic MAUI tool developed internationally by the EuroQoL group, has shown excellent psychometric properties across a wide range of population, conditions and settings [28]. It has been widely used in COVID-19 patients after discharge in different countries of the world [29]. An advantage of this instrument over others is that it has a short form consisting of only five questions. Therefore, it allows a rapid survey of HRQoL among participants. As we planned to interview patients coming for follow-up in a relatively busy setting, we planned to use the EQ-5D-5L instrument in this study. The EQ-5D-5L instrument [30] explores five dimensions of health, including mobility, self-care, usual activities, pain/discomfort, and anxiety/depression, with five questions as well as overall health rated on a visual analog scale (EQ VAS). It is a self-completed instrument. Each question has five levels of response, corresponding to “no problem”, “slight problem”, “moderate problem”, “severe problem” and “unable to do a particular activity or extreme problem”. The EQ VAS score ranges from 0 (worst health) to 100 (best health). The validated Bangla version of the EQ-5D-5L provided by Euroqol group [23] was used for this study. The questionnaire was explained to the patient and then given to the patient to complete by themselves or with assistance from the interviewer if the participant is unable to read.

### Data collection procedure

Physicians were trained and placed in the COVID-19 post-acute care and follow-up clinic of DMCH for data collection. All consecutive patients coming for post-acute care and consenting for enrollment were included. Sociodemographic information was obtained from the discharge certificate.

### Anthropometric measurements

Participants’ body-weight in light clothes was measured to the nearest 0.1 kg using a properly calibrated mechanical bathroom weight scale (Tanita mechanical weight scale, model: HA680, manufactured in Japan). A locally-manufactured, measuring tape was used to measure height of participants to the nearest 0.5 cm. During measurement of height, it was ensured that patients stood upright on a flat surface without shoes and with back of the heels and the occiput remaining on the wall. BMI was calculated using the standard formula. BMI was categorized as underweight (<18.5 kg/m^2^), normal (18.5–22.9 kg/m^2^), overweight (23–24.99 kg/m^2^) and obese (≥ 25 kg/m^2^) based on the proposed classification for Asian adults by WHO [31].

### Statistical analysis

A single utility score was calculated using US values sets of EQ-5D (ranging from -0.109 to 1.000) [32], as no values sets were available for the general population of Bangladesh or the neighboring countries. Descriptive statistics was used to describe participant characteristics in frequency (percentage) or mean (±SD). Categorical variables were described by frequency and percentage. Continuous variables were reported as the mean(±SD) or median (range) where appropriate. Analytic statistics were applied to test association between HRQoL and factor variables. The normality of continuous variables was checked. Then, independent samples t-test and one-way analysis of variance (ANOVA) were used to compare continuous variables across groups. Associations between categorical data were tested using the chi-square test. Multivariable logistic regression analysis was done to explore factors associated with slight to extreme problem in five dimensions of HRQoL separately. Factors which came significant at <0.05 level were considered for the multivariable logistic analysis. All tests were two-tailed, and p values <0.05 were considered statistically significant. The internal consistency of the EQ-5D-5L instrument for our dataset was checked by Cronbach’s alpha (coefficient = 0.8755). The statistical software Stata (version 16) was used for data analysis.

### Ethical consideration

All procedures were conducted following the ethical guidelines of the institution’s Ethical Review Committee (ERC) of Dhaka Medical College Hospital (ERC-DMC/ECC/2021/434 date:26/12/21). The ethical standards laid down in the 1964 Declaration of Helsinki and its later amendments or comparable ethical standards were followed wherever applicable. Informed written consent was obtained from the patients or their guardians when critically ill before enrollment in the study.

## Results

A total of 563 participants were included in the final analysis. The average age of the participants was 51.18 (±13.49) years (SD). Of them, 30.09% were aged 50–59 years, and 30.45% were aged ≥60 years. The majority of the participants were male (55.95%), living in urban areas (63.86%), and nonsmokers (73.90%). The most frequent occupation was a housewife (39.02%). The average BMI of the participants was 25.25 (4.62) kg/m2 (SD), and the majority (44.06%) were overweight. Out of all, 61.10% had at least one comorbidity. The mean EQ-5D-5L index score (±SD) was 0.78 (± 0.19), and the EQ VAS score was 70.26 (±11.13). Sixty and above aged participants had a significantly lower index score than those aged 18–39 years (p<0.05). Similarly, the EQ VAS score was also significantly lower in the older age group (50–59 and ≥60 years) than in the younger age group (18–39 years, p<0.05). Both index and EQ VAS scores were significantly lower in females than in males (p<0.001). Participants who were retired had the lowest mean score on the EQ-5D-5L index and EQ VAS component (p<0.001) among the occupation groups. Housewives also had a lower index and EQ VAS score in comparison to other occupation groups (p<0.001). Those living in rural and peri-urban areas had lower index and EQ VAS scores than those living in urban areas (p = 0.009 and p = 0.004, respectively.). Overweight participants had a higher mean index and EQ VAS score than participants from other BMI categories (p<0.001 for both). Individuals with presence of (at least one) symptom had a significantly lower index and EQ VAS score (p<0.001). No significant difference was noted in index and EQ VAS scores in relation to the smoking habit, presence of comorbidities, and severity of COVID-19 (p>0.05 for all) (**Table 1**).

**Table 1:** Characteristics of respondents and EQ-5D index and visual analogue (EQ VAS) score.

Characteristics	n (%)	EQ-5D-5L Index		EQ-5D-5L VAS	
		Mean (SD)	p-value	Mean (SD)	p-value
**Total**	563	0.78 (0.19)		70.26 (11.13)	
**Age (years), Mean (SD)**	51.18 (13.49)				
**Age groups**					
18–29	29 (5.23)	0.87 (0.14)	<0.001	77.06 (8.40)	<0.001
30–39	84 (15.14)	0.84 (0.18)		73.86 (9.29)	
40–49	106 (19.10)	0.79 (0.17)		69.88 (10.77)	
50–59	167 (30.09)	0.78 (0.18)		69.19 (10.30)^a^^,^^b^	
≥60	169 (30.45)	0.73 (0.22)^a^^,^^b^		68.86 (12.73)^a^^,^^b^	
**Sex**					
Male	315 (55.95)	0.81 (0.01)	<0.001	71.82 (11.92)	<0.001
Female	248 (44.05)	0.75 (0.01)		68.31 (9.70)	
**Occupation**					
Housewife	199 (39.02)	0.74 (0.19)	<0.001	67.26 (8.66)	<0.001
Job Holder	83 (16.27)	0.85 (0.14)^a^		72.95 (10.59)^a^	
Businessman	57 (11.18)	0.85 (0.15)^a^		71.58 (9.96)^a^	
Others	94 (18.43)	0.83 (0.16)^a^		74.18 (10.58)^a^	
Retired	77 (15.10)	0.71 (0.23)^b^^,^^c^^,^^d^		66.87 (12.77)^c^^,^^d^	
**Setting**					
Urban	341 (63.86)	0.80 (0.19)	0.009	71.22 (11.26)	0.004
Rural	65 (12.17)	0.75 (0.18)^a^		68.00 (12.02)	
Peri-urban	128 (23.97)	0.75 (0.17)		67.97 (8.57)^a^	
**Body Mass Index (kg/m** ^**2**^ **), Mean (SD)**	25.25 (4.62)				
**Body Mass Index Categories (kg/m** ^**2**^ **)**					
Underweight (<18.5)	36 (8.39)	0.67 (0.21)	<0.001	64.72 (14.24)	<0.001
Normal (18.5–22.9)	81 (18.88)	0.75 (0.19)		67.71 (10.78)	
Overweight (23.0–27.5)	189 (44.06)	0.82 (0.17)^a^^,^^b^		72.74 (9.86)^a^^,^^b^	
Obese (>27.5)	123 (28.67)	0.75 (0.19)^c^		69.02 (10.36)^c^	
**Smoking habit**					
Nonsmoker	371 (73.90)	0.78 (0.20)	0.673	69.94 (10.88)	0.158
Ex-smoker	109 (4.38)	0.78 (0.18)		67.75 (10.10)	
Current smoker	22 (21.71)	0.81 (0.17)		70.45 (10.46)	
**Comorbidities**					
Present	344 (61.10)	0.76 (0.19)	<0.001	68.76 (10.95)	<0.001
Absent	219 (38.90)	0.83 (0.19)		72.64 (11.01)	
**Symptom**					
Present	517 (91.83)	0.77 (0.19)	<0.001	69.64 (10.80)	<0.001
Absent	46 (8.17)	0.88 (0.19)		77.34 (12.29)	
**COVID-19 severity** ^!^					
**Moderate**	548 (97.34)	0.78 (0.01)	0.285	70.23 (0.47)	0.623
**Severe**	15 (2.66)	0.73 (0.05)		71.67 (3.37)	

*p*-value was determined by independent samples t-test and one-way ANOVA where appropriate. Pairwise comparisons were done using Tukey (p-value significant at <0.05 level in comparison to first category^a^,

second category^b^,

third category^c^

and fourth category^d^.)

Proportion was calculated after excluding missing values.

^!^Severity of COVID-19 at admission to hospital during acute illness: Mild disease CVID19 with SPO_2_ > 90% and no radiological pneumonia, Moderate disease–there is radiological evidence of pneumonia but oxygen saturation more than 90%, and severe disease–where oxygen saturation equal to or less than 90%.

Overall, 45.77%, 50.99%, 52.79%, 55.14%, and 62.16% had problems in mobility, self-care, usual activities, pain/discomfort, and anxiety/depression dimensions, respectively (**Table 2)**. Elderly participants (≥60 years) had the highest proportion of problems in mobility, self-care, and pain/discomfort dimensions (p<0.001, p = 0.005, p = 0.001, respectively). Problems in usual activities were faced mostly by 50- to 59-year-old participants (p = 0.003). Anxiety/depression related problems were the most common in the 40- to 49-year-old group (p = 0.012). Female participants showed a significantly higher proportion of problems than males in all dimensions (p = 0.012 for mobility and p<0.001 for the rest of the dimensions). Housewives had the highest proportion of problems in the usual activities, pain/discomfort, and anxiety/depression dimensions (p<0.001 for all). On the other hand, retired participants had the highest proportion of problems in mobility and self-care domains (p<0.001 for all). Participants living in peri-urban areas had the most frequently reported problems in mobility, self-care, and anxiety/depression dimensions (p = 0.001, p = 0.002, and p = 0.001, respectively). A significantly higher proportion of problems in the pain/discomfort domain was described by participants living in rural areas (p = 0.001). Participants who were underweight, had comorbidities and symptoms had a significantly higher proportion of problems in all dimensions (p<0.05 for all comparisons). Proportion of responses across all five levels in five dimensions of EQ-5D-5L is given in **S1 to S5 Tables in S1 File**. **S6 and S7 Tables in S1 File** enlist the comorbidities and clinical features of the participants at follow-up one after recovery from COVID-19. The two most common comorbidities were diabetes mellitus (40.5%) and hypertension (30.2%). Cough (33.93%) and dyspnea (26.64%) were present in more than one-quarter of the participants. The average SpO2 at rest was 97.25% (SD 2.74).

**Table 2:** Proportion of participants reporting problems in five dimensions of EQ-5D-5L.

Characteristics	Mobility			Self-care			Usual activities			Pain/discomfort			Anxiety/depression		
	No	Slight to extreme	*p value*	No	Slight to extreme	*p value*	No	Slight to extreme	*p value*	No	Slight to extreme	*p value*	No	Slight to extreme	*p value*
**Total**	54.23	45.77		49.01	50.99		47.21	52.79		44.86	55.14		37.84	62.16	
**Age (years) (%)**															
18–29	75.86	24.14	<0.001	65.52	34.48	0.005	65.52	34.48	0.003	65.62	34.48	0.001	41.38	58.62	0.012
30–39	66.67	33.33		61.90	38.10		61.90	38.10		60.71	39.29		54.76	45.24	
40–49	56.60	43.40		49.06	50.94		49.06	50.94		43.40	56.60		32.08	67.92	
50–59	54.49	45.51		49.10	50.90		41.32	58.68		41.92	58.08		35.33	64.67	
≥60	42.60	57.40		39.64	60.36		41.42	58.58		37.28	62.72		34.91	65.09	
**Sex (%)**															
Male	59.37	40.63	0.012	57.46	42.54	<0.001	56.51	43.49	<0.001	52.70	47.30	<0.001	46.35	53.64	<0.001
Female	48.79	51.21		39.11	60.89		35.48	64.52		35.08	64.92		28.23	71.77	
**Occupation (%)**															
Housewife	45.73	54.27	<0.001	34.67	65.33	<0.001	34.17	65.83	<0.001	32.16	67.84	<0.001	24.62	75.38	<0.001
Job Holder	68.67	31.33		65.06	34.94		63.86	36.14		59.04	40.96		49.40	50.60	
Businessman	64.91	35.09		64.91	35.09		59.65	40.35		56.14	43.86		56.14	43.86	
Others	64.89	35.11		60.64	39.36		55.32	44.68		54.26	45.74		45.74	54.26	
Retired	41.56	58.44		32.47	67.53		38.96	61.04		40.26	59.74		28.57	71.43	
**Setting (%)**															
Urban	60.12	39.88	0.001	53.67	46.33	0.002	49.56	50.44	0.161	49.56	50.44	0.002	42.82	57.18	0.001
Peri-urban	40.00	60.00		36.72	63.28		39.84	60.16		37.50	62.50		24.22	75.78	
Rural	46.09	53.91		40.00	60.00		44.62	55.38		29.23	70.77		35.38	64.62	
**BMI (kg/m** ^**2**^ **) (%)**															
Underweight (<18.5)	30.56	69.44	0.003	33.33	66.67	0.015	25.00	75.00	<0.001	22.22	77.78	<0.001	25.00	75.00	0.010
Normal (18.5–22.9)	54.32	45.68		43.21	56.79		37.04	62.96		30.86	69.14		28.40	71.60	
Overweight (23.0–27.5)	61.38	38.62		53.97	46.03		56.61	43.39		55.03	44.97		45.50	54.50	
Obese (>27.5)	47.97	52.03		38.21	61.79		35.77	64.23		38.21	61.79		33.33	66.67	
**Smoking habit (%)**															
Nonsmoker	54.18	45.82	0.983	47.17	52.83	0.692	45.82	54.18	0.476	43.67	56.33	0.833	35.31	64.69	0.523
Ex-smoker	53.21	46.79		50.46	49.54		45.87	54.13		43.12	56.88		41.28	58.72	
Current smoker	54.55	45.45		54.55	45.45		59.09	40.91		50.00	50.00		36.36	63.64	
**Comorbidities (%)**															
Present	49.14	50.86	0.001	42.73	57.27	<0.001	41.28	58.72	<0.001	37.21	62.79	<0.001	31.69	68.31	<0.001
Absent	63.47	36.53		59.82	40.18		56.62	43.38		57.08	42.92		48.86	51.14	
**Symptom (%)**															
**Present**	53.00	47.00	0.006	47.20	52.80	0.001	45.07	54.93	0.001	42.36	57.64	<0.001	35.20	64.80	<0.001
**Absent**	73.91	26.09		73.91	26.09		71.74	28.26		73.91	26.09		73.91	26.09	
**COVID severity**^!^ **(%)**															
Moderate	55.47	44.53	0.025	49.27	50.73	0.756	47.63	52.37	0.306	45.44	54.56	0.149	38.14	61.86	0.503
Severe	26.67	73.33		53.33	46.67		33.33	66.67		26.67	73.33		46.67	53.33	

*p*-value was determined by Chi-square test or Fisher’s exact test where appropriate; Data represents row percentage; BMI: Body Mass Index.

^!^ Severity of COVID-19 at admission to hospital during acute illness: Mild disease–COVID-19 with SPO_2_ > 90% and no radiological pneumonia, Moderate disease–there is radiological evidence of pneumonia but oxygen saturation more than 90%, and Severe disease–where oxygen saturation equal to or less than 90%.

Multivariable logistic regression analysis included all the above-mentioned factors except smoking and COVID-19 severity (except mobility dimension) to determine independent factors associated with problems in the five dimensions of the EQ-5D-5L scale **(Table 3)**. In mobility dimensions, ≥60 age (odds ratio [OR] 3.24, 95% confidence interval [CI]: 1.07–9.77), rural and peri-urban settings (OR 2.26, 95% CI: 1.09–4.28, and OR 1.89, 95% CI: 1.13–3.20, respectively) were significantly associated with problems. Being retired (OR 2.57, 95%CI: 1.06–6.25), being overweight (OR 0.53, 95% CI 0.29–0.98) and presence of symptom (OR 3.27, 95%CI 1.31–8.18) were independent factors associated with problems in self-care. Age ≥60 years (OR 3.11, 95%CI: 1.02–9.50), Female sex (OR 5.50, 95% CI: 2.22–13.62) and being overweight (OR 0.42, 95% CI: 0.23–0.78) were associated with problems in usual activities. Participants living in peri-urban areas were 1.82 times (95% CI 1.04–3.12) more likely than those living in urban areas to have problems in pain/discomfort dimensions. Similarly, those with comorbidities and symptoms were, respectively, 1.75 times (95% CI: 1.07–2.85) and 3.08 times (95%CI: 1.21–7.86) more likely than those without comorbidities and symptom to have problems in pain/discomfort. On the other hand, overweight individuals were 70% less likely (95% CI: 0.16–0.57) to have similar problems. In the anxiety/depression domains, retired individuals (OR 6.21, 95% CI: 2.37–16.27), peri-urban settings (OR 2.16, 95% CI: 1.22–3.84) and those having symptoms (OR 3.35, 95%CI: 1.35–8.30) were more likely than businessmen, urban settings and those without symptoms to have problems, respectively. Also, obese individuals were significantly less likely (OR 0.47, 95%CI: 0.25–0.90) than those with normal BMI to have problems.

**Table 3:** Multivariable logistic regression analysis to determine factors associated with slight to extreme responses in the five dimensions of the EQ-5D.

Characteristics	Mobility		Self-care		Usual activities		Pain/discomfort		Anxiety/depression	
	OR (95%CI)	*p value*	OR (95%CI)	*p value*	OR (95%CI)	*p value*	OR (95%CI)	*p value*	OR (95%CI)	*p value*
**Age (years)**										
18–29	Ref		Ref		Ref		Ref		Ref	
30–39	1.04 (0.35–3.06)	0.949	0.73 (0.25–2.10)	0.560	0.57 (0.19–1.69)	0.315	0.84 (0.29–2.46)	0.752	0.45 (0.15–1.31)	0.141
40–49	1.43 (0.48–4.23)	0.517	1.39 (0.48–4.01)	0.547	1.19 (0.40–3.51)	0.751	1.26 (0.43–3.70)	0.674	0.77 (0.26–2.30)	0.***642***
50–59	1.41 (0.49–4.04)	0.524	1.29 (0.46–3.60)	0.631	2.05 (0.72–5.89)	0.180	1.40 (0.49–3.97)	0.531	0.86 (0.30–2.47)	0.775
≥60	3.24 (1.07–9.77)	**0.037**	2.17 (0.73–6.48)	0.164	3.11 (1.02–9.50)	**0.046**	2.89 (0.94–8.83)	0.064	0.84 (0.27–2.57)	0.759
**Sex**										
Male	Ref		Ref		Ref		Ref		Ref	
Female	2.24 (0.95–5.31)	0.066	2.16 (0.92–5.07)	0.078	5.50 (2.22–13.62)	**<0.001**	2.15 (0.90–5.11)	0.084	2.27 (0.97–5.35)	0.060
**Occupation**										
Businessman	Ref		Ref		Ref		Ref		Ref	
Housewife	1.19 (0.39–3.61)	0.755	1.92 (0.64–5.77)	0.246	0.61 (0.20–1.91)	0.399	2.18 (0.71–6.68)	0.174	2.21 (0.73–6.69)	0.159
Job Holder	1.31 (0.54–3.15)	0.546	1.15 (0.49–2.73)	0.745	0.93 (0.39–2.23)	0.876	1.39 (0.58–3.34)	0.458	1.72 (0.74–4.02)	0.211
Others	1.22 (0.51–2.89)	0.652	1.35 (0.58–3.15)	0.486	1.08 (0.45–2.55)	0.867	1.51 (0.64–3.57)	0.352	1.57 (0.68–3.64)	0.295
Retired	1.86 (0.78–4.46)	0.162	2.57 (1.06–6.25)	**0.037**	1.19 (0.49–2.86)	0.702	1.45 (0.60–3.54)	0.413	6.21 (2.37–16.27)	**<0.001**
**Setting**										
Urban	Ref		Ref		Ref		Ref		Ref	
Rural	2.16 (1.09–4.28)	**0.028**	1.71 (0.84–3.47)	0.140	1.15 (0.57–2.33)	0.697	1.79 (0.86–3.73)	0.117	1.53 (0.74–3.17)	0.249
Peri-Urban	1.89 (1.13–3.20)	**0.016**	2.67 (1.22–3.63)		1.58 (0.92–2.72)		1.82 (1.04–3.12)	**0.036**	2.16 (1.22–3.84)	**0.008**
**BMI (kg/m** ^**2**^ **)**										
Underweight (< 18.5)	2.44 (0.96–6.19)	**0.060**	1.34 (0.51–3.51)	0.548	1.88 (0.69–5.10)	0.218	1.23 (0.47–3.59)	0.615	1.15 (0.40–3.26)	0.800
Normal (18.5–22.9)	Ref				Ref		Ref		Ref	
Overweight (23.0–27.5)	0.82 (0.45–1.48)	0.507	0.53 (0.29–0.98)	**0.042**	0.42 (0.23–0.78)	**0.006**	0.30 (0.16–0.57)	**<0.001**	0.47 (0.25–0.90)	**0.024**
Obese (>27.5)	1.32 (0.69–2.55)	0.403	0.89 (0.45–1.75)	0.728	1.03 (0.52–2.06)	0.912	0.49 (0.24–0.99)	0.047	0.65 (0.32–1.32)	0.236
**Comorbidities**										
Absent	Ref		Ref		Ref		Ref		Ref	
Present	1.32 (0.82–2.13)	0.248	1.45 (0.89–2.35)	0.132	1.40 (0.86–2.28)	0.177	1.75 (1.07–2.85)	**0.026**	1.63 (0.99–2.68)	0.052
**Symptom**										
Absent	Ref		Ref		Ref		Ref		Ref	
Present	2.10 (0.85–2.12)	0.248	3.27 (1.31–8.18)	**0.011**	3.08 (1.21–7.87)	**0.018**	2.75 (1.09–6.96)	**0.032**	3.35 (1.35–8.30)	**0.009**
COVID-19 severity*										
Moderate	Ref		-		-		-		-	
Severe	1.43 (0.31–6.63)		-		-		-		-	

*COVID-19 severity was included in mobility dimension only because it did not come significant in bivariate analysis for other dimensions;

Significant p-values were shown in bold.

BMI: Body mass index

## Discussion

Several studies have assessed COVID-19 patients’ HRQoL during acute illness, during follow-up after discharge from the hospital, and after recovery in non-hospitalized cases by different MAUIs.

[13, 15]. The commonly used instruments are the Short Form Health Survey 36 (SF-36) [26], EQ-5D-5L [30], and WHOQOL BREF [14], and CDC HRQOL-14 [27]. There is no single ‘gold standard’ for measuring the quality of life [25]. The WHOQOL BREF instrument assess HRQOL in four domains including physical, psychological, social and environmental domains using a set of 25 questions [14]. The SF-36 probes eight domains focusing physical, psychological and social aspects of health [26] with a set of 36 questions. The CDC HRQOL-14 and EQ-5D-5L instruments mainly focuses on the physical and psychological dimensions of HRQOL [27, 32] using a set of 14 and 5 questions, respectively. Here, the former instrument gives a measure of healthy days spent over last month. On the other hand, the EQ-5D-5L allows a rapid survey of HRQOL because of its short structure. Previously, the CDC HRQOL-14 instrument [16] and WHOQOL BREF [15] were used in COVID-19 patients who completed one month and a median 172 days after being tested positive, respectively, in population-based settings. However, we targeted to include only hospitalized COVID-19 patients one month after discharge in post-acute-care and follow-up clinic, and therefore, we intended to use a rapidly deployable instrument for HRQOL. Hence, the EQ-5D-5L instrument was used.

The average EQ-5D index score (SD) was 0.78 (±0.19), and the average EQ VAS score (SD) was 70.26 (±11.13) in our patients during follow-up at the first month. Which is higher than that found in south-central Ethiopia [20]. They found an average index score of 0.68 (±0.28) among the hospitalized COVID-19 patients after discharge. In contrast, an Italian study, doing HRQOL assessment a median 61 days after discharge from ICU, found that the mean EQ VAS score was 74, higher than that of the present study. Similarly, another study in Brazil [33] found that the mean index score was 0.80, three-months after discharge from the hospital. The differences in score might be due to differences in regional socio-economic context and time passed since discharge.

Our analysis revealed that the HRQOL was significantly lower in patients who were older (≥60 years) and had comorbidities, which is evident from many previous studies [15, 29]. Older people are more likely to have comorbidities, so a lower QoL can be expected. However, after adjustment for comorbidities and other factors, compared to younger age (18–29 years), ≥60 age was found to be associated with a higher chance of having problems in mobility only. The study by Islam and colleagues [16] found that comorbidity was significant associated with patient’s need for help for routine needs. Whereas we found that comorbidities were independently associated with increased pain/discomfort, which might explain why these people needs help with their routine activity.

Female patients were more likely to have a significantly lower index and EQ VAS score than males, which similar to the findings of previous studies which used the EQ-5D scale [34, 35]. By the same token, housewives and retired individuals had a lower EQ-5D index and EQ VAS score than jobholders, businessmen, and others. However, in the multivariable logistic regression, female patients showed a statistically significant problem in usual activities, and retired individuals had problems in self-care. In Bangladesh, the majority of women spend most of their time in domestic activities, and most households survive on their daily household chores [36]. Hence, female patients emphasizing their problems in usual activities imply that COVID-19 was probably hampering their daily routine and disrupting the family balance. Interestingly, Kaso and colleagues did not find such sex-based differences in EQ-5D index scores during the acute illness due to COVID-19 [20], suggesting that during the acute illness, surviving the disease was the most important matter at hand. Persistence of physical weakness more in female than male patients after being discharged from the hospital, as observed by Qu and colleagues [18], could add to the explanation of why female patients had more problems in usual activity during acute post-care in our study.

Elderly people who are engaged in activities find less difficulty in self-care than those who are not engaged [37]. This could explain why retired COVID-recovered persons had the most problems in self-care in this study. However, as elderly people often require assistance in their care and as the highly contagious COVID-19 made isolation a norm for the prevention of spread [38], finding people for assistance in the time of COVID-19 could be a difficult task in itself.

Our study found that people living in peri-urban settings were the most sufferers in terms of quality of life in comparison to those living in urban settings. Even after adjustment for other determinants, patients coming from peri-urban areas were significantly more likely to report problems in mobility, self-care, pain/discomfort, and anxiety/depression. This could be due to the overall unhealthiness of the peri-urban community. A previous study by Debnath and Debnath found that the peri-urban areas were the least healthier area compared to urban and rural areas in terms of composite health measures [39].

We did not find any difference in index score or EQ VAS in relation to the severity of COVID-19 during the acute illness among patients, probably due to the small sample size of severe cases. However, counterintuitively, we found that overweight patients, classified according to WHO Asian criteria [31], were significantly less likely than normal-weight patients to have problems in self-care, usual activities, pain/discomfort, and anxiety/depression. As other factors are working in concert, this finding lends support to the idea that obesity might not be the main driver of QoL [40].

Islam and colleagues [16] reported that nearly four-fifth (86%) patients had various type of symptoms and the presence of symptoms, one-month after diagnosis of COVID-19, was significantly associated with poor/fair general health condition and requirement of help with daily needs. Our findings if nearly similar in that we found more than ninety percent (91.83%) patients having various type of symptoms, and presence of symptom was significantly associated with problems in self-care, usual activities, pain/discomfort and anxiety/depression. In explanation, we agree with the arguments made by Islam and colleagues [16] that symptoms might have caused increased illness feeling and morbidity severity leading to hindrance with activities, care and mental health problems.

### Limitation and strength

The study was limited in that it was a single-center study. Investigations could not be done due to limited resources. A strong association between HRQOL and its determinants could be inferred because of the cross-sectional nature of the study. However, the strength of the study was that it conducted a face-to-face interview of the participants rather than using telephonic interview, and the sample size was adequate for exploring interaction between factors and HRQOL.

## Conclusions

The present study found that the HRQOL of COVID-19 patients one month after discharge in different dimensions was significantly associated with age, sex, occupation, setting, body mass index, presence of symptoms and comorbidities. Based on our study findings we recommend that specific management strategies tailored to address the factors should be planned to improve the health-related quality of life of COVID-19 patients after discharge.

## Supporting information

S1 File Supplementary tables. (PDF)

S2 File Dataset. (SAV)
