# Supplementary material for: Post-discharge quality of life of COVID-19 patients at 1-month follow-up: A cross-sectional study in the largest tertiary care hospital of Bangladesh
Source: PLoS One. 2023 Jan 31;18(1):e0280882. doi: 10.1371/journal.pone.0280882 (PMC9888719; doi:10.1371/journal.pone.0280882)
Supplement: S1 File — (PDF) [file pone.0280882.s001.pdf]

S1 Table. Proportion of participants reporting problems in mobility dimension of EQ-5D-5L

	Mobility				
	**No problem**	**Slight problem**	**Moderate problem**	**Severe problem**	**Unable to walk**
**Total, n(%)**	301 (54.71)	214 (38.01)	36 (6.39)	4 (0.71)	1 (0.18)
**Age (years), n(%)**					
18 – 29	22 (75.86)	7 (24.14)	0 (0.00)	0 (0.00)	0 (0.00)
30 – 39	56 (66.67)	23 (27.38)	4 (4.76)	1 (1.19)	0 (0.00)
40 – 49	60 (56.60)	39 (36.79)	6 (5.66)	1 (0.94)	0 (0.00)
50 – 59	91 (54.49)	66 (39.52)	9 (5.39)	1 (0.60)	0 (0.00)
≥ 60	72 (42.60)	78 (46.15)	17 (10.06)	1 (0.59)	1 (0.59)
**Sex, n(%)**					
Male	187 (59.37)	104 (33.02)	22 (6.98)	1 (0.32)	1 (0.32)
Female	121 (48.79)	110 (44.35)	14 (5.65)	3 (1.21)	0 (0.00)
**Occupation, n(%)**					
Housewife	91 (4.73)	96 (48.24)	10 (5.03)	2 (1.01)	0 (0.00)
Job Holder	57 (68.67)	26 (31.33)	0 (0.00)	0 (0.00)	0 (0.00)
Businessman	37 (64.91)	17 (29.82)	3 (5.26)	0 (0.00)	0 (0.00)
Others	61 (64.89)	27 (28.72)	6 (6.38)	0 (0.00)	0 (0.00)
Retired	32 (41.56)	34 (44.16)	10 (12.99)	0 (0.00)	1 (1.30)
**Setting, n(%)**					
Urban	205 (60.12)	112 (32.84)	21 (6.16)	2 (0.59)	1 (0.29)
Rural	26 (40.00)	34 (52.31)	5 (7.69)	0 (0.00)	0 (0.00)
Semi-urban	59 (46.09)	62 (48.44)	6 (4.69)	1 (0.78)	0 (0.00)
**BMI (kg/m^2), n(%)**					
Underweight (< 18.5)	11 (30.56)	18 (50.00)	7 (19.44)	0 (0.00)	0 (0.00)
Normal (18.5 – 22.9)	44 (54.32)	32 (39.51)	5 (6.17)	0 (0.00)	0 (0.00)
Overweight (23.0 – 27.5)	116 (61.38)	66 (34.92)	7 (3.70)	0 (0.00)	0 (0.00)
Obese (>27.5)	59 (47.97)	51 (41.46)	10 (8.13)	3 (2.44)	0 (0.00)
**Smoking habit, n(%)**					
Non-smoker	201 (54.18)	145 (39.08)	22 (5.93)	3 (0.81)	0 (0.00)
Ex-smoker	12 (53.98)	43 (39.45)	7 (6.42)	0 (0.00)	1 (0.92)
Current smoker	12 (54.55)	10 (45.45)	0 (0.00)	0 (0.00)	0 (0.00)
**Comorbidities, n(%)**					
Present	169 (49.13)	146 (42.44)	25 (7.27)	3 (0.87)	1 (0.29)
Absent	139 (63.47)	68 (31.05)	11 (5.02)	1 (0.46)	0
**Symptom, n(%)**					
At least one	274 (53.00)	204 (39.46)	32 (6.58)	4 (0.77)	1 (0.19)
None	34 (73.91)	10 (21.74)	2 (4.35)	0 (0.00)	0 (0.00)

**COVID severity, n(%)**					
Moderate	304 (55.47)	205 (37.41)	34 (6.20)	4 (0.73)	1 (0.18)
Severe	4 (26.67)	9 (60.00)	2 (13.33)	0 (0.00)	0 (0.00)

The number within parenthesis indicates row percentage. Percentage was calculated after exclusion of missing values.

S2 Table. Proportion of participants reporting problems in self-care dimension of EQ-5D-5L

	**Self-care**				
	**No problem**	**Slight problem**	**Moderate problem**	**Severe problem**	**Unable to wash or dress**
**Total, n(%)**	278 (49.38)	218 (38.72)	55 (9.77)	6 (1.07)	6 (1.07)
**Age (years), n(%)**					
18 – 29	19 (65.52)	9 (31.03)	1 (3.45)	0 (0.00)	0 (0.00)
30 – 39	52 (61.90)	30 (35.71)	1 (1.19)	1 (1.19)	0 (0.00)
40 – 49	52 (49.06)	46 (43.40)	6 (5.66)	0 (0.00)	2 (1.89)
50 – 59	82 (49.10)	64 (38.32)	19 (11.38)	1 (0.60)	1 (0.60)
≥ 60	67 (39.64)	67 (39.64)	28 (16.57)	4 (2.37)	3 (1.78)
**Sex, n(%)**					
Male	181 (57.46)	93 (29.52)	33 (10.48)	4 (1.27)	4 (1.27)
Female	97 (39.11)	125 (50.40)	22 (8.87)	2 (0.81)	2 (0.81)
**Occupation, n(%)**					
Housewife	69 (34.67)	107 (53.77)	19 (9.55)	2 (1.01)	2 (1.01)
Job Holder	54 (65.06)	24 (28.92)	5 (6.02)	0 (0.00)	0 (0.00)
Businessman	37 (64.91)	17 (29.82)	3 (5.26)	0 (0.00)	0 (0.00)
Others	57 (60.64)	31 (32.98)	6 (6.38)	0 (0.00)	0 (0.00)
Retired	25 (32.47)	29 (37.66)	18 (23.38)	3 (3.90)	2 (2.60)
**Setting, n(%)**					
Urban	183 (53.67)	122 (35.78)	29 (8.50)	2 (0.59)	5 (1.47)
Rural	26 (40.00)	31 (47.69)	7 (10.77)	1 (1.54)	0 (0.00)
Semi-urban	47 (36.72)	61 (47.66)	18 (14.06)	1 (0.78)	1 (0.78)
**BMI (kg/m^2), n(%)**					
Underweight (< 18.5)	12 (33.33)	15 (41.67)	7 (19.44)	1 (2.79)	1 (2.78)
Normal (18.5 – 22.9)	35 (43.21)	32 (39.51)	11 (13.58)	1 (1.23)	2 (2.47)
Overweight (23.0 – 27.5)	102 (53.97)	75 (39.68)	10 (5.29)	1 (0.53)	1 (0.53)
Obese (>27.5)	47 (38.21)	61 (49.59)	13 (10.57)	1 (0.81)	1 (0.81)
**Smoking habit, n(%)**					
Non-smoker	175 (47.17)	152 (40.97)	36 (9.70)	5 (1.35)	3 (0.81)
Ex-smoker	55 (50.46)	38 (34.86)	14 (12.84)	1 (0.92)	1 (0.92)
Current smoker	12 (54.55)	9 (40.91)	1 (4.55)	0 (0.00)	0 (0.00)
**Comorbidities, n(%)**					
Present	147 (42.73)	144 (41.86)	46 (13.37)	4 (1.16)	3 (0.87)
Absent	131 (59.82)	74 (33.79)	9 (4.11)	2 (0.91)	3 (1.37)
**Symptom, n(%)**					
At least one	244 (47.20)	210 (40.62)	53 (10.25)	5 (0.97)	5 (0.97)
None	34 (73.91)	8 (17.39)	2 (4.35)	1 (2.17)	1 (2.17)

**COVID severity, n(%)**					
Moderate	270 (49.27)	213 (38.87)	54 (9.85)	6 (1.09)	5 (0.91)
Severe	8 (53.33)	5 (33.33)	1 (6.67)	0 (0.00)	1 (6.67)

The number within parenthesis indicates row percentage. Percentage was calculated after exclusion of missing values.

S3 Table. Proportion of participants reporting problems in usual-activity dimension of EQ-5D-5L

	Usual activity				
	**No problem**	**Slight problem**	**Moderate problem**	**Severe problem**	**Unable to do usual activity**
**Total, n(%)**	266 (47.25)	254 (45.12)	34 (6.04)	6 (1.07)	3 (0.53)
**Age (years), n(%)**					
18 – 29	19 (65.52)	10 (34.48)	0 (0.00)	0 (0.00)	0 (0.00)
30 – 39	52 (61.90)	29 (34.52)	3 (3.57)	0 (0.00)	0 (0.00)
40 – 49	52 (49.05)	46 (43.40)	6 (5.66)	0 (0.00)	2 (1.89)
50 – 59	69 (41.32)	84 (50.30)	12 (7.19)	1 (0.60)	1 (0.60)
≥ 60	70 (41.42)	81 (47.93)	13 (7.69)	5 (2.96)	0 (0.00)
**Sex, n(%)**					
Male	178 (56.51)	12 (35.56)	19 (6.03)	3 (0.95)	3 (0.95)
Female	88 (35.48)	142 (57.26)	15 (6.05)	3 (1.21)	0 (0.00)
**Occupation, n(%)**					
Housewife	68 (34.17)	166 (58.29)	12 (6.03)	3 (1.51)	0 (0.00)
Job Holder	53 (63.86)	29 (34.94)	1 (1.20)	0 (0.00)	0 (0.00)
Businessman	34 (59.65)	21 (36.84)	2 (3.51)	0 (0.00)	0 (0.00)
Others	52 (55.32)	40 (42.55)	2 (2.13)	0 (0.00)	0 (0.00)
Retired	30 (38.96)	35 (45.45)	9 (11.69)	3 (3.90)	0 (0.00)
**Setting, n(%)**					
Urban	169 (49.56)	152 (44.57)	16 (4.69)	2 (0.59)	2 (0.59)
Rural	29 (44.62)	28 (43.08)	7 (10.77)	1 (1.54)	0 (0.00)
Semi-urban	51 (39.84)	67 (52.34)	9 (7.03)	1 (0.78)	0 (0.00)
**BMI (kg/m^2), n(%)**					
Underweight (< 18.5)	9 (25.00)	19 (52.78)	6 (16.67)	2 (5.56)	0 (0.00)
Normal (18.5 – 22.9)	30 (37.04)	42 (51.85)	7 (8.64)	1 (1.23)	1 (1.23)
Overweight (23.0 – 27.5)	107 (56.61)	76 (40.21)	5 (2.65)	0 (0.00)	1 (0.53)
Obese (>27.5)	44 (35.77)	68 (55.28)	9 (7.32)	2 (1.63)	0 (0.00)
**Smoking habit, n(%)**					
Non-smoker	170 (45.82)	170 (45.82)	26 (7.01)	4 (1.08)	1 (0.27)
Ex-smoker	50 (45.87)	54 (49.54)	3 (2.75)	2 (1.83)	0 (0.00)
Current smoker	13 (59.09)	9 (40.91)	0 (0.00)	0 (0.00)	0 (0.00)
**Comorbidities, n(%)**					
Present	142 (41.28)	170 (49.42)	27 (7.85)	5 (1.42)	0 (0.00)
Absent	124 (56.62)	84 (38.36)	7 (3.20)	1 (0.46)	3 (1.37)
**Symptom, n(%)**					
At least one	233 (45.07)	245 (47.39)	31 (6.00)	5 (0.97)	3 (0.58)

None	33 (71.74)	9 (19.57)	3 (6.52)	1 (2.17)	0 (0.00)
**COVID severity, n(%)**					
Moderate	261 (47.63)	246 (44.89)	33 (6.02)	5 (0.91)	3 (0.55)
Severe	5 (33.33)	8 (53.33)	1 (6.67)	1 (6.67)	0 (0.00)

The number within parenthesis indicates row percentage. Percentage was calculated after exclusion of missing values.

S4 Table. Proportion of participants reporting problems in pain/discomfort dimension of EQ-5D-5L

	**Pain/discomfort***			
	**No**	**Slight**	**Moderate**	**Severe**
**Total, n(%)**	253 (44.94)	257 (45.65)	45 (7.99)	8 (1.42)
**Age (years), n(%)**				
18 – 29	19 (65.52)	10 (34.48)	0 (0.00)	0 (0.00)
30 – 39	51 (60.71)	29 (34.52)	3 (3.57)	1 (1.19)
40 – 49	46 (43.40)	51 (48.11)	9 (8.49)	0 (0.00)
50 – 59	70 (41.92)	82 (49.10)	14 (8.38)	1 (0.60)
≥ 60	63 (37.28)	81 (47.93)	19 (11.24)	6 (3.55)
**Sex, n(%)**				
Male	166 (52.70)	121 (38.41)	23 (7.30)	5 (1.59)
Female	87 (35.08)	136 (54.84)	22 (8.87)	3 (1.21)
**Occupation, n(%)**				
Housewife	64 (32.16)	114 (57.29)	18 (9.05)	3 (1.51)
Job Holder	49 (59.04)	32 (38.55)	2 (2.41)	0 (0.00)
Businessman	32 (56.14)	23 (40.35)	2 (3.51)	0 (0.00)
Others	51 (54.26)	36 (38.30)	7 (7.45)	0 (0.00)
Retired	31 (40.26)	33 (42.86)	9 (11.69)	4 (5.19)
**Setting, n(%)**				
Urban	169 (49.56)	147 (43.11)	21 (6.16)	4 (1.17)
Rural	19 (29.23)	35 (53.85)	10 (15.38)	1 (1.54)
Semi-urban	48 (37.50)	68 (53.13)	11 (8.59)	1 (0.78)
**BMI (kg/m^2), n(%)**				
Underweight (< 18.5)	8 (22.22)	20 (55.56)	7 (19.44)	1 (2.78)
Normal (18.5 – 22.9)	25 (30.86)	47 (58.02)	8 (9.88)	1 (1.23)
Overweight (23.0 – 27.5)	104 (55.03)	74 (39.15)	11 (5.82)	0 (0.00)
Obese (>27.5)	47 (38.21)	64 (52.03)	10 (8.13)	2 (1.63)
**Smoking habit, n(%)**				
Non-smoker	162 (43.67)	170 (45.82)	32 (8.63)	7 (1.89)
Ex-smoker	47 (43.12)	54 (49.54)	7 (6.42)	1 (0.92)
Current smoker	11 (50.0)	9 (40.91)	2 (9.09)	0 (0.00)
**Comorbidities, n(%)**				
Present	128 (37.21)	174 (50.58)	36 (10.47)	6 (1.74)
Absent	125 (57.08)	83 (37.90)	9 (4.11)	2 (0.91)
**Symptom, n(%)**				
At least one	219 (42.36)	250 (48.36)	40 (7.74)	8 (1.55)

None	34 (73.91)	7 (15.22)	5 (10.87)	0 (0.00)
**COVID severity, n(%)**				
Moderate	249 (45.44)	249 (45.44)	42 (7.66)	8 (1.46)
Severe	4 (26.67)	8 (53.33)	3 (20.00)	0 (0.00)

The number within parenthesis indicates row percentage. Percentage was calculated after exclusion of missing values. * The extreme category was excluded as there were no response.

S5 Table. Proportion of participants reporting problems in anxiety/depression dimension of EQ-5D-5L

	**Anxiety/ Depression**			
	**No**	**Slight**	**Moderate**	**Severe**
**Total, n(%)**	266 (47.25)	254 (45.12)	34 (6.04)	6 (1.07)
**Age (years), n(%)**				
18 – 29	12 (41.38)	17 (58.62)	0 (0.00)	0 (0.00)
30 – 39	46 (54.76)	34 (40.48)	3 (3.57)	1 (1.19)
40 – 49	34 (32.08)	67 (63.21)	5 (4.72)	0 (0.00)
50 – 59	59 (35.33)	96 (57.49)	12 (7.19)	0 (0.00)
≥ 60	59 (34.91)	95 (56.21)	14 (8.28)	1 (0.59)
**Sex, n(%)**				
Male	146 (46.35)	151 (47.94)	18 (5.71)	0 (0.00)
Female	70 (28.23)	160 (64.52)	16 (6.45)	2 (0.81)
**Occupation, n(%)**				
Housewife	49 (24.62)	139 (69.85)	10 (5.03)	1 (0.50)
Job Holder	41 (49.40)	35 (42.17)	7 (8.43)	0 (0.00)
Businessman	32 (56.14)	25 (43.86)	0 (0.00)	0 (0.00)
Others	43 (45.74)	45 (47.87)	5 (5.32)	1 (1.06)
Retired	22 (28.57)	48 (62.34)	7 (5.09)	0 (0.00)
**Setting, n(%)**				
Urban	146 (42.82)	171 (50.15)	22 (6.45)	2 (0.59)
Rural	23 (35.38)	38 (58.46)	4 (6.15)	0 (0.00)
Semi-urban	31 (24.22)	90 (70.31)	7 (5.47)	0 (0.00)
**BMI (kg/m^2), n(%)**				
Underweight (< 18.5)	9 (25.00)	19 (52.78)	8 (22.22)	0 (0.00)
Normal (18.5 – 22.9)	23 (28.40)	51 (62.96)	7 (8.64)	0 (0.00)
Overweight (23.0 – 27.5)	86 (45.50)	96 (50.79)	6 (3.17)	1 (0.53)
Obese (>27.5)	41 (33.33)	74 (60.16)	7 (5.69)	1 (0.81)
**Smoking habit, n(%)**				
Non-smoker	131 (35.31)	217 (58.49)	21 (5.66)	2 (0.54)
Ex-smoker	45 (41.28)	57 (52.29)	7 (6.42)	0 (0.00)
Current smoker	8 (36.36)	12 (54.55)	2 (9.09)	0 (0.00)
**Comorbidities, n(%)**				
Present	109 (31.69)	210 (61.05)	23 (6.69)	2 (0.58)
Absent	107 (48.86)	101 (46.12)	11 (5.02)	0 (0.00)
**Symptom, n(%)**				
At least one	182 (35.20)	301 (58.22)	32 (6.19)	2 (0.39)
None	34 (73.91)	10 (21.74)	2 (4.35)	0 (0.00)

**COVID severity, n(%)**				
Moderate	209 (38.14)	303 (55.29)	34 (6.20)	2 (0.36)
Severe	7 (46.67)	8 (53.33)	0 (0.00)	0 (0.00)

The number within parenthesis indicates row percentage. Percentage was calculated after exclusion of missing values. * The extreme category was excluded as there were no response.

S6 Table. Comorbidities of participants (n=563)

**Comorbidities**	**n (%)**
Diabetes Mellitus	228 (40.50)
Hypertension	170 (30.20)
Bronchial Asthma	42 (7.46)
Ischemic Heart Disease	36 (6.39)
Hypothyroidism	27 (4.80)
Chronic Kidney Disease	21 (3.73)
Chronic Obstructive Pulmonary Disease	15 (2.66)
Stroke	5 (0.89)
Parkinsonism	1 (0.18)
Osteoarthritis of knee	1 (0.18)
Myelofibrosis	1 (0.18)

S7 Table. Clinical features of participants (n=563)

**Characteristics**	
**Symptoms**	**n (%)**
Cough	191 (33.93)
Dyspnoea	150 (26.64)
Chest pain	130 (23.09)
Fatigue	128 (22.74)
Anorexia	121 (21.49)
Sleep disturbances	105 (18.65)
Fever	69 (12.26)
Palpitation	58 (10.30)
Joint pain	41 (7.28)
Headache	39 (6.93)
Bodyache	27 (4.80)
Limb pain	28 (4.97)
Vertigo	26 (4.62)
Weight loss	17 (3.02)
Skin rash	16 (2.84)
Abdominal pain	15 (2.66)
Dizziness	8 (1.42)
Altered bowel habit	6 (1.07)
Dysgeusia	9 (1.60)
Dysosmia	6 (1.07)
Vomiting	3 (0.53)
Hemoptysis	2 (0.36)
Sensory disturbance	2 (0.36)
Convulsion	2 (0.36)
Hair loss	2 (0.36)
Hearing loss	2 (0.36)
**Signs**	Mean (SD)
Pulse (beats/min)	92.49 (13.99)
Systolic blood pressure (mmHg)	122.25 (18.92)
Diastolic blood pressure (mmHg)	79.27 (11.79)
Temperature (F)	98.04 (0.98)
SpO_2 at rest	97.25 (2.74)
